# Effects of Zinc Glycinate on Growth Performance, Serum Biochemical Indexes, and Intestinal Morphology of Yellow Feather Broilers

**DOI:** 10.1007/s12011-021-02990-x

**Published:** 2021-11-05

**Authors:** Xiaoping Zhu, Xiuguo Shang, Guozhao Lin, Haojie Li, Xin Feng, Huihua Zhang

**Affiliations:** School of Life Science and Engineering, Foshan University, Foshan, 528231 China

**Keywords:** Zinc glycinate, Yellow feather broiler, Growth performance, Serum index, Intestinal morphology, Metallothionein

## Abstract

The purpose of this study was to investigate the effects of zinc glycinate (Gly-Zn) on growth performance, serum biochemical index, intestinal morphology, and hepatic metallothionein (MT) mRNA expression in the liver of yellow feather broilers. A total of 540 18-day-old yellow feather broilers were randomly divided into three groups: control group (basal diet), ZnSO_4_ group (basal diet plus 60 mg Zn/kg from ZnSO_4_), and Gly-Zn group (basal diet plus 60 mg Zn/kg from zinc glycinate). Each treatment group had 6 replicates with 30 birds in each replicate. The experiment lasted for 42 days (18 to 59 days of age). The results showed that Gly-Zn supplementation significantly improved the average daily gain (ADG) and average daily feed intake (ADFI) of broilers during 18 to 39 days of age compared with that in the control group (*P* < 0.05) but not different from the ZnSO_4_ group. The Gly-Zn group had higher glutathione peroxidase (GSH-Px) (*P* < 0.05) and lower malondialdehyde (MDA) concentrations than the broilers in the control and ZnSO4 group. It was also observed that zinc content in the tibia of Gly-Zn group broilers was higher than the control and ZnSO4 group (*P* < 0.05). The results of intestinal morphology parameters showed that the Gly-Zn group significantly increased the villus height in duodenum and jejunum (*P* < 0.05) and decreased crypt depth in duodenum and ileum compared to the control group. However, there were no significant differences between the Gly-Zn group and ZnSO_4_ group in duodenum and ileum regarding intestinal morphology parameters. The Gly-Zn group significantly increased mRNA expression of MT in the liver than both control and ZnSO_4_ groups (*P* < 0.05). Collectively, the results indicated that supplementing 60 mg Zn/kg through zinc glycinate improved growth performance and serum indexes as well as intestinal morphology of yellow feather broilers. It also regulates MT gene expression more effectively than the ZnSO_4_ group at the transcriptional level.

## Introduction

Zinc is a cofactor for more than 240 endogenous enzymes involved in the metabolism of proteins, lipids, carbohydrates, and nucleic acids. Animal growth, reproduction, immune regulation, and hormonal activity are all related to zinc [1–6]. NRC (1994) recommended that the amount of zinc added to broiler diets was 40 mg/kg. Zinc is usually supplemented in the form of sulfate in animal diets due to the relatively low cost. However, the bioavailability of zinc in plant feed ingredients and conventional inorganic zinc sources (such as zinc sulfate and zinc oxide) is poor [7]. Furthermore, excessive addition of inorganic zinc in diets can cause serious environmental pollution.

Many studies have shown that the organic form of trace elements could prevent it from producing indigestible complexes with certain anti-nutritional compounds and mineral antagonist in the intestine [8]. Huang et al. [9, 10] showed that organic zinc has higher bioavailability than inorganic sources. Organic Zn could reduce negative environmental impact by decreasing manure zinc excretion. Amino acid chelated zinc could improve the performance of broilers and intestinal morphology, and optimize cecal microflora [11–13]. Metallothionein (MT) plays an important role in metabolism regulation and its expression is regulated by zinc level in the diet [9, 10]. It can bind with Zn^2+^ and Cu^2+^ in plasma and be released when the body needs it. Therefore, the level of MT mRNA expression can be used as an effective marker to evaluate the zinc status in the body [11]. Additionally, Rao et al. found that supplementation of organic forms of Zn increased body mass gain and feed intake of broiler chicken compared to those fed the control diet [12]. However, limited information regarding whether organic Zn (zinc glycinate) regulates the growth performance of yellow feather broilers.

Therefore, the present study was set up to investigate the effects of zinc glycinate on the growth performance, intestinal morphology, and antioxidant status of yellow feather broilers. The findings of this study would provide valuable information for the selection and application of zinc supplements in broiler production.

## Materials and Methods

### Diets and Zinc Sources

The experiment diet was a corn-soybean–based pellet diet (Foshan Guangmuxing Feed Co., Ltd., Foshan, China), and the nutritional level of the diet for each stage refers to the nutritional requirements of Chinese yellow feather broiler (NY/T 33–2004). The diet composition and nutrient information are shown in Table 1. Zinc sulfate monohydrate (Zn, 34.5%) and zinc glycinate (Zn, 21%) were provided by Guangdong Xingtengke Biotechnology Co., Ltd. (Foshan, China).

**Table 1:** Composition and nutrient levels of the basal diet (air-dry basis, %)

Ingredients	18–39 days	40–59 days	Nutrient levels^2)^	18–39 days	40–59 days
Corn	63.00	66.00	ME/(MJ/kg)	12.76	13.37
Soybean meal	28.00	21.50	CP	19.00	17.00
Corn gluten meal	2.00	3.00	Lys	1.10	1.00
Wheat middling	0.17	0.23	Met	0.50	0.45
Lard oil	3.00	5.50	Zn ( mg/kg)	31.00	28.00
Limestone	1.32	1.32			
Calcium hydrophosphate	1.33	1.33			
DL-Met	0.18	0.12			
Premix^1)^	1.00	1.00			
Total	100.00	100.00			

^1)^The premix provided the following per kg of diets: VA 12 000 IU, VD_3_ 5 000 IU, VB_2_ 5 mg, VK_3_ 2 mg, VE 30 mg, VB_1_ 3 mg, VB_12_ 1 mg, nicotinic acid 3 g, pantothenic acid 800 mg, folic acid 500 mg, biotin 0.2 mg, choline 500 mg, Fe 10 g, Cu 8 g, Mn 10 g, I 42 mg, Se 30 mg

^2)^Zn was a measured value, while the others were calculated values

### Experimental Design, Sampling, and Laboratory Analysis

A total of 540 healthy (18 days old) broilers were randomly divided into three groups: control group (basal diet), ZnSO_4_ group (basal diet plus 60 mg Zn/kg from ZnSO_4_), and Gly-Zn group (basal diet plus 60 mg Zn/kg from zinc glycinate). There were six replicates per group and 30 chickens per replicate. All groups of chickens were kept under the same conditions with 23-h lighting from incandescent bulbs and free access to feed and water. The experiment lasted for 42 days. Before the study, the chicken house, cage, and feed trough were rinsed out and then disinfected. The temperature and relative humidity were controlled within the range of 24–26 °C and 85 to 90%, respectively. The health status of the birds was monitored and the disease and mortality were recorded.

The body weight of broilers was measured at 18, 40, and 59 days of age, and average daily feed consumption was determined. The average daily feed intake (ADFI), average daily gain (ADG), and feed-to-weight ratio (F/G) were calculated. At 40 and 59 days of age, eighteen chickens from each group were randomly selected and 4 mL of the blood was collected from the wing vein. The blood samples were centrifuged at 3000 g/min for 10 min, and the supernatant was collected for later analysis. The albumin, total protein, malondialdehyde (MDA), and glutathione peroxidase (GSH-Px) were measured by enzyme-linked immunosorbent assay kits provided by Nanjing Jiancheng Bioengineering Research Institute (albumin, A028-2–1; total protein, A045-2–2; MDA, A003-1–2; GSH-Px, A005-1–2; Nanjing, China).

On the last day of the trial, three broilers were randomly selected from each replicate and slaughtered. The segments of duodenum, jujunum, and ileam were collected (1 cm). The intestinal samples were rinsed with cold phosphate buffer solution and fixed in 4% formaldehyde solution for 24 h, then embedded with paraffin, sliced, and stained. The length of the villi and the depth of the crypt were observed microscopically and measured.

The liver samples were sampled and stored at – 80 °C for later determination of expression of the hepatic metallothionein (MT-1) gene. Total RNA was extracted (TransGen Biotechnology Co., Ltd., Dalian, China) and 1 μg RNA was reverse-transcribed into cDNA using the PrimeScript RT Reagent Kit (TaKaRa Biotechnology, Dalian, China) according to the manufacturer’s guidelines. Quantitative real-time polymerase chain reaction (qRT-PCR) was performed on an ABI StepOnePlus™ Real-Time PCR System (Applied Biosystems, Grand Island, NY, USA). The sequence of a primer used in this experiment was referred to Varum et al. [13] (forward 5′-AAG GGC TGT GTC TGC AAG GA-3′, reverse 5′-CTT CAT CGG TAT GGA AGG TAC AA-3′) and was synthesized by Shanghai Bioengineering Technology Co., Ltd (Shanghai, China). The relative mRNA expressions of target genes were calculated using the 2^−∆∆Ct^ method as previously reported [14].

Two chickens were selected for each repeat 2 days before weighing. The excreta and daily feed intake of the selected chickens within 48 h were accurately recorded. Collect chicken manure every 2 h, remove feathers, dandruff, and other sundries, and then put it into the dung box for weighing. Collection of ileal contents at slaughter on the last day of the trial. The ileal contents and excreta samples collected from each chicken were dried at 65 °C for 48–72 h, then recovered at room temperature for 24 h, and crushed. The tibia, intestinal segment, and feces were crushed, dried at 65 °C for 12 h, dried at 105 °C to constant weight, returned to moisture at room temperature for 12 h, and then crushed again. We weigh 2.0 g of the crushed sample into a crucible, carbonize the sample to smokeless in an electric furnace, cool it to normal temperature, then ash it in a high-temperature electric furnace at 500–550 °C for 8 h, cool it to normal temperature, add 2 mL of 1 mol/L nitric acid, bake in an oven at 35 °C overnight, and then transfer the solution to a 50-mL volumetric flask for constant volume. The content of zinc in the sample was determined by Ice-3500 atomic absorption spectrometry (Thermo Fisher Scientific, Waltham, USA).

### Statistical Analysis

The data was analyzed by using the one-way ANOVA in SPSS (24.0). The treatment effects were included in the model as fixed effects. Multiple comparisons were performed using Duncan’s method. The difference was declared significant at *P* < 0.05 and highly significant at *P* < 0.01. The results were expressed as “mean ± mean standard error.”

## Results and Discussion

### Growth Performance

The effect of zinc supplementation on the growth performance of yellow feather broilers is presented in Table 2. At 40 days of age, the broilers from the Gly-Zn groups had higher body weight compared to the control groups but not different from the ZnSO4 group broilers. The average daily gain (ADG) and average daily feed intake (ADFI) of Gly-Zn group broilers were significantly higher than that of the control group between 18 and 39 (*P* < 0.05), but had no difference compared with the ZnSO_4_ group. The feed-to-weight ratio (F/W) of the Gly-Zn group was lower than the control group during the whole study period but not different from that of the ZnSO_4_ group.

**Table 2:** Effects of zinc supplementation on the growth performance of yellow-feathered broilers

		Control	ZnSO_4_	Gly-Zn	SEM	*P*-value
BW (40 days)		818.21^b^	822.22^ab^	840.83^a^	6.72	0.042
BW (59 days)		1486.80	1488.59	1526.35	16.43	0.083
18–39 days	ADFI (g)	54.05^b^	54.67^ab^	56.19^a^	0.06	0.032
	ADG (g)	26.39^b^	26.54^ab^	27.41^a^	0.03	0.025
	F/W	2.04	2.05	2.05	0.01	0.084
40–59 days	ADFI (g)	83.17	82.78	83.30	0.46	0.156
	ADG (g)	35.18	35.07	36.07	0.54	0.183
	F/W	2.36	2.36	2.31	0.03	0.261
18–59 days	ADFI (g)	68.99	69.05	70.28	1.46	0.336
	ADG (g)	30.35	30.59	31.42	1.02	0.421
	F/W	2.27^a^	2.25^ab^	2.22^b^	0.01	0.023

There are three treatments, control, a basal diet; ZnSO4, a basal diet plus ZnSO4; Gly-Zn, a basal diet plus zinc glycinate. *n* = 6/treatment. ^a,b,c^Values labeled with different superscripts in the same row differ significantly (*p* < 0.05)

Zinc can promote the feed intake and weight gain of animals by promoting the rapid proliferation of taste bud cells in tongue mucosa, prolonging the residence time of feed in the digestive tract, and improving the secretion of the digestive system and the activity of enzymes in tissue cell [15, 16]. Many studies have shown that organic zinc is more effective than inorganic zinc in improving the feed intake and daily gain of broilers, shown by reduced feed conversion rate and improved economic benefits. However, in our study, there was no difference detected between the inorganic zinc group (ZnSO_4_) and the zinc glycinate (Gly-Zn) group. Aoyagi and Baker [17] reported that corn-soybean-type diets supplemented with lysine zinc significantly increased feed conversion in broilers. Star et al. [18] found that compared with inorganic zinc, organic zinc would significantly increase the feed intake of hens and reduce the feed-to-weight ratio. But, these were not observed in our study. This might be caused by the difference in breeds, diet, and environment.

### Blood Parameters and Zinc Content

The effects of zinc supplementation on blood parameters and Zn concentration of yellow feather broilers are presented in Table 3. The serum total protein and albumin of the Gly-Zn group was significantly higher than the control group (*P* < 0.05), but not different from the ZnSO_4_ group. Serum albumin was increased by 28.28% (*P* < 0.05) in the Gly-Zn group compared with the control group at 59 days of age. The Gly-Zn group had a lower concentration of MDA than both the control and ZnSO_4_ group but higher in terms of Zn content in the tibia (*P* < 0.05). The Gly-Zn group significantly improved the concentration of Zn in feces and ileum contents compared with the control group (*P* < 0.05). Higher serum total protein content indicates that the body’s protein metabolism was well balanced, which was beneficial to the absorption and utilization of protein [19, 20]. Kucuk et al. [21] reported that supplement with ZnSO_4_ significantly increased the protein concentration in the serum of broilers. Our results were consistent with Kucuk’s findings. Yu et al. [22] added organic zinc (zinc methionine) and inorganic zinc (zinc sulfate) to broiler diets and found that the plasma total zinc and albumin contents in the organic zinc group were higher than those in the inorganic zinc groups. Abedini et al. [23] showed that the supplementation with Zn significantly affected serum total protein and albumin concentrations in comparison with the control. Prasad [24] reported that Zn deficiency may cause abnormalities in nucleic acid synthesis and the activity of many enzymes. In the present study, our results showed that supplemented with Zn increased serum proteins, but there was no difference between organic zinc and inorganic zinc on serum protein.

**Table 3:** Effects of zinc supplementation on serum biochemical indexes and zinc concentration in tissue of yellow-feathered broilers

Items	Control			ZnSO_4_		Gly-Zn		SEM		*P*-value
40 days										
Serum total protein (g/L)	36.88^b^			37.46^ab^		39.95^a^		0.72		0.028
Serum albumin (g/L)	14.96			15.00		16.00		0.61		0.412
GSH-Px (U/mL)	310.85^b^			311.49^b^		341.32^a^		8.34		0.019
MDA (nmoL/mL)	7.84^a^			7.65^a^		6.65^b^		0.32		0.037
Zinc concentration in tissues (mg/kg)										
Tibia	341.00^b^			354.35^b^		380.95^a^		7.65		0.012
Feces	286.03^b^			298.54^b^		324.47^a^		9.22		0.028
Ileum	20.43^b^			23.09^a^		23.85^a^		1.26		0.035
59 days										
Serum total protein (g/L)	27.70^b^			30.40^ab^		33.90^a^		1.85		0.021
Serum albumin (g/L)	11.03^b^			13.01^ab^		14.15^a^		0.81		0.014
GSH-Px (U/mL)	322.79^b^			332.18^ab^		342.12^a^		5.46		0.016
MDA (nmoL/mL)	4.21			4.01		3.82		0.38		0.365
Zinc concentration in tissues (mg/kg)										
Tibia		384.70^c^		414.00^b^		432.00^a^		5.32		0.007
Feces		343.85^a^		352.52^ab^		362.95^b^		5.28		0.038
Ileum		28.62^c^		34.01^b^		39.74^a^		1.38		0.027

There are three treatments, control, a basal diet; ZnSO4, a basal diet plus ZnSO4; Gly-Zn, a basal diet plus zinc glycinate. *n* = 6/treatment. ^a,b,c^Values labeled with different superscripts in the same row differ significantly (*p* < 0.05)

GSH-Px is an enzyme that removes H_2_O_2_ and organic peroxides from living organisms, prevents lipid peroxides from damaging body tissues and biofilms, and averts from further hydrolysis of lipid peroxides into harmful substances MDA. The MDA concentration in the body can indirectly reflect the oxygen free radical metabolism in the body, the degree of free radical attack of the body tissue cells, and the degree of lipid peroxidation. Many studies have explored the relationship between zinc and antioxidant indexes. A study by De Grande et al. [16] showed that supplementation with Zn-AA complexes alleviated oxidative stress indicated by decreased MDA plasma levels and GSH-Px activities. Previous studies indicated that zinc source and zinc levels significantly affected GSH-Px activity and effectively reduced MDA content [25–27]. Ma et al. [11] suggested that zinc supplementation could decrease MDA levels in the liver compared to the control group, which confirms the importance of zinc supplementation in broilers. Fathi et al. [28] suggested that different zinc sources had different effects on the antioxidant activity of broilers by regulating the GSH-Px and MDA contents. Similarly, the results of the current study showed that organic zinc supplementation significantly improved GSH-Px content and decreased MDA content in the early stage of the yellow-feathered broiler.

Zinc supplement clearly increases fecal Zn excretion and Zn concentration ileum content in our study, which is consistent with previous studies’ results [29]. Zinc can improve bone formation by stimulating cell proliferation, collagen synthesis in osteoblastic cells, and mineralization [30–32]. Zn had also an effect on the mechanical properties of bones [33]. Bone strength and bone zinc content were easily affected by the amount of zinc added to the diet. Sandoval et al. [34] found that the zinc content of the tibia increased with the zinc levels of supplementation. Shelton and Southern reported that the breaking strength of the tibia increased significantly over 14 days on a diet supplemented with 75 ppm Zn [35]. In addition, the content of zinc in the bone was influenced by the types of zinc added to the diets. Kwiecień et al. observed that the addition of Zn-Gly increased the accumulation of Zn both in the tibia and in the femur of the birds compared with Zn-SO_4_ [36]. In a study investigating the bioavailability of ZnSO_4_ and methionine zinc, the experiment results showed that the zinc content of the tibia of broilers in the methionine zinc group was higher than that in the ZnSO_4_ group [37]. Wedekind et al. [38] also found that zinc methionine could significantly increase the zinc content of the cavity bone compared with ZnSO_4_ and ZnO. Alkhtib et al. [39] reported a significant increase in tibia strength associated with feeding the chelated zinc or M-Nano-Zn supplements compared with ZnSO_4_ at 21 days post-hatch*.* In our study, the Gly-Zn group had higher tibia Zn content compared with the ZnSO_4_ group indicating that the organic zinc might have higher bioavailability. According to the present study, the use of Zn-Gly contributed to improving the quality of the tibia and the femur and their strength parameters, perhaps due to the increased deposition of Ca and P in bones.

### Intestinal Morphology

The effects of zinc supplementation on small intestine morphology and structure of yellow feather broilers are presented in Table 4. The villi height and ratio of villus height to crypt depth of duodenum and jejunum of the Gly-Zn group were higher than the control group but no difference compared with the ZnSO_4_ group (*P* < 0.05). In the ileum, the Gly-Zn group had lower crypt depth and a higher ratio of villus height to crypt depth compared to the control group and the ZnSO_4_ groups (*P* < 0.05).

**Table 4:** Effects of zinc supplementation on intestinal morphology of yellow-feathered broilers

Item		Control	ZnSO_4_	Gly-Zn	SEM	*P*-value
Duodenum	Villus height (μm)	1377.40^b^	1448.66^ab^	1477.27^a^	29.22	0.036
	Crypt depth (μm)	223.02^a^	194.05^b^	193.97^b^	8.26	0.021
	V/C^1)^	6.29^b^	7.59^ab^	8.02^a^	0.51	0.019
Jejunum	Villus height (μm)	1112.96^b^	1139.15^ab^	1174.09^a^	16.46	0.028
	Crypt depth (μm)	175.91	162.94	157.20	11.26	0.241
	V/C	6.40^b^	7.04^ab^	7.59^a^	0.33	0.039
Ileum	Villus height (μm)	1172.34	1179.77	1247.95	32.11	0.279
	Crypt depth (μm)	204.33^a^	208.44^a^	150.00^b^	12.67	0.034
	V/C	6.04^b^	6.65^b^	8.53^a^	0.62	0.023

There are three treatments, control, a basal diet; ZnSO4, a basal diet plus ZnSO4; Gly-Zn, a basal diet plus zinc glycinate. *n* = 6/treatment. ^a,b,c^Values labeled with different superscripts in the same row differ significantly (*p* < 0.05)

^1)^V/C means villus height/crypt depth ratio

The small intestine is an important location for the absorption of nutrients in the animal’s body. The villus height of the small intestine and the crypt depth are important indicators for assessing the digestion and absorption capacity of the small intestine. Ewtushik et al. [40] had shown that with the villus height of the small intestine increasing, the number of epithelial cells was also increased, and the ability to absorb nutrients in the gastrointestinal tract enhanced. The depth of crypts reflects the maturity and rate of increase of epithelial cells. As the crypts become shallower, the nutrient absorption capacity is enhanced. The ratio between villus height and crypt depth reflects the absorption capacity of the small intestine, and the absorption capacity of the small intestine increases as the ratio increases [41]. Park et al. [42] found that dietary zinc supplementation would effectively improve the intestinal villus height of weaned piglets, reduce the depth of crypts, and improve health condition. De Grande et al. [16] showed that compared with inorganic zinc, zinc amino acid in broilers’ feed was more easily absorbed and could protect villous epithelial cells at the initial stage. On the 10th and 28th days, the villus length of broilers supplemented with zinc amino acid complex significantly increased. Li et al. [43] results indicated that 80 mg/kg Zn-Met supplementation increased villus height, villus area, and villus height/crypt depth ratio and reduced crypt depth in the jejunum of laying hens compared with the control and ZnSO4 group. In this study, the Gly-Zn group broiler had higher villus height and lower crypt depth in the duodenum and jejunum than the control group broilers but was not different from the ZnSO_4_ group broilers. In the ileum, the Gly-Zn group broiler had lower crypt depth and a higher ratio of villus height to crypt depth compared to the control and ZnSO_4_ group, which indicated that the superiority of organic zinc than the inorganic zinc source.

### MT mRNA Expression

**Fig. 1:**
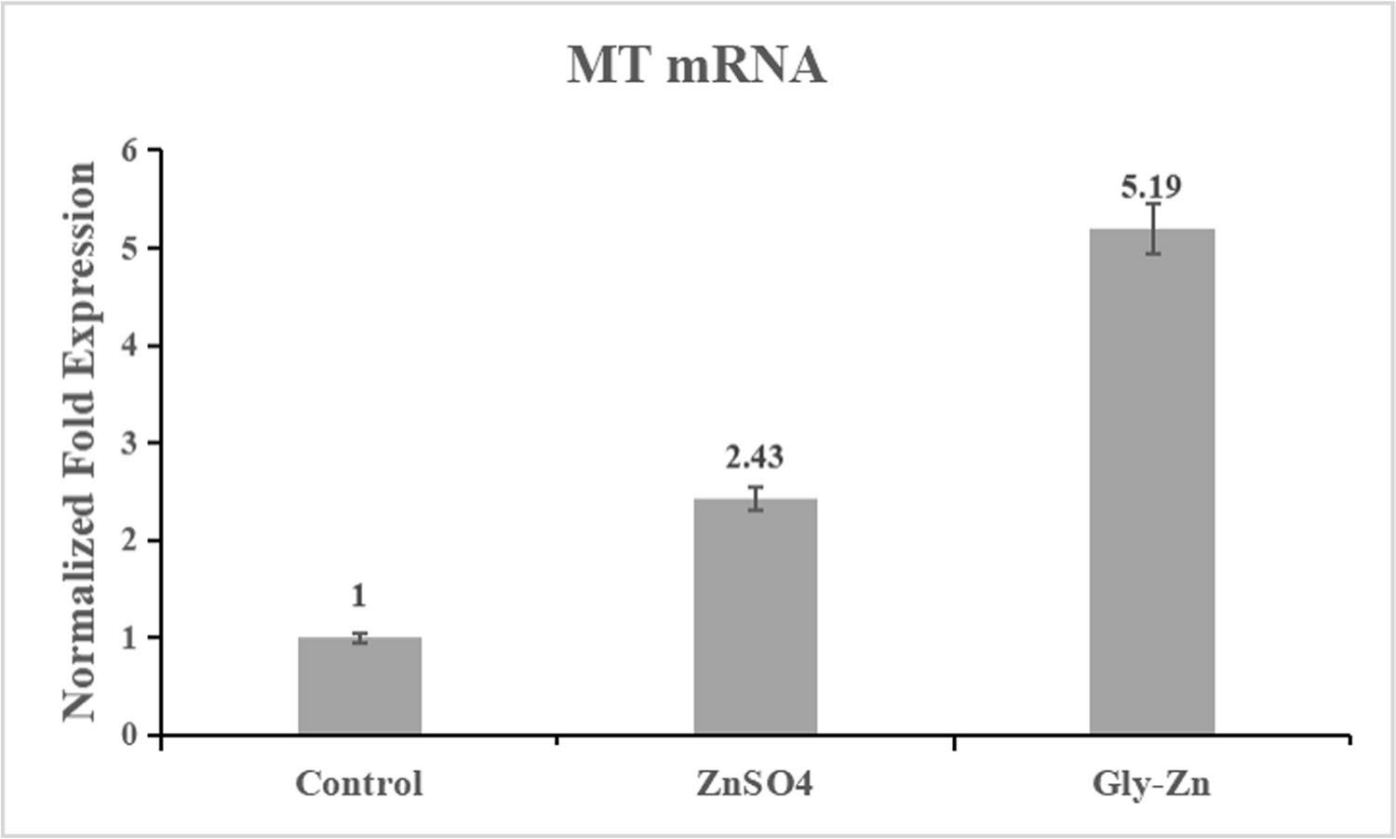
Effects of different zinc supplementation on MT mRNA expression in livers of yellow-feathered broilers. There are three treatments, control, a basal diet; ZnSO4, a basal diet plus ZnSO4; Gly-Zn, a basal diet plus zinc glycinate; *n* = 6/treatment

The effects of different zinc supplementations on MT mRNA expressions in livers of yellow feather broilers are presented in Fig. 1. The hepatic MT mRNA expression of the Gly-Zn group was significantly higher than the control group and ZnSO_4_ group (*P* < 0.05). Zinc can effectively regulate MT gene expression at the transcriptional level [44–46]. Wang et al. [47] found that MT concentrations in the liver and pancreas of broilers supplemented with organic zinc were significantly higher than those in the control group. Different zinc sources also have an important effect on the expression of MT mRNA. Cao et al. [48] also showed that organic Zn sources with moderate or strong complex strength slightly upregulated the MT protein level in the intestine of broilers, compared with the inorganic Zn source. A previous study showed that on day 28, metallothionein (MT) mRNA levels in the duodenum, jejunum, and ileum were enhanced (*P* < 0.05) with Zn addition regardless of Zn source [49]. These were also observed in the current study as there were significant differences between the Gly-Zn group and the ZnSO_4_ group, which indicated that organic zinc could have more effective regulation for MT gene expression than inorganic zinc at the transcriptional level.

## Conclusions

Supplementation of 60 mg Zn/kg in the form of zinc glycinate to the corn-soybean–based diet resulted in better growth performance of the yellow-feathered broilers. Compared to ZnSO_4_, zinc glycinate improved the antioxidant status, increased the tibia Zn content, and had more effective regulation for MT gene expression at the transcriptional level.

## Data Availability

The data used to support the findings are all included in the article.
